# Preterm Labor and Hypertensive Disorders in Adolescent Pregnancies With Diabetes Between 2006 and 2019

**DOI:** 10.1155/2024/2283730

**Published:** 2024-10-18

**Authors:** Estelle Everett, Christina S. Han, Michael Richley, Timothy P. Copeland, Tannaz Moin, Lauren E. Wisk

**Affiliations:** 1 Division of Endocrinology, Diabetes, and Metabolism, Department of Medicine, David Geffen School of Medicine, University of California, Los Angeles, California, USA; 2 Division of General Internal Medicine and Health Services Research, Department of Medicine, David Geffen School of Medicine, University of California, Los Angeles, California, USA; 3 Department of Medicine, VA Greater Los Angeles Healthcare System, Los Angeles, California, USA; 4 Division of Maternal Fetal Medicine, Department of Obstetrics and Gynecology, David Geffen School of Medicine, University of California, Los Angeles, California, USA; 5 Division of Maternal Fetal Medicine, Department of Obstetrics and Gynecology, University of Washington School of Medicine, Seattle, Washington, USA; 6 Division of Nephrology, Department of Medicine, University of California, San Francisco, California, USA; 7 Department of Health Policy and Management, Fielding School of Public Health, University of California, Los Angeles, California, USA

## Abstract

**Objective:**

We sought to evaluate the risk of preterm labor and hypertensive disorders in adolescent pregnancies with and without diabetes.

**Methods:**

We evaluated 1,843,139 adolescents (≤20 years old) with labor and delivery admissions in the national Kids’ Inpatient Database (KID) in years 2006, 2009, 2012, 2016, and 2019. International classification of disease codes was used to identify diabetes and medical factors affecting pregnancy. Weighted logistic regression was used to evaluate the association between diabetes and complications.

**Results:**

Among admissions, 0.2% had type 1 diabetes (T1D), 0.2% had type 2 diabetes (T2D), and 0.7% had gestational diabetes (GDM); 10.1% of admissions were complicated by hypertensive disorders and 5.8% by preterm labor. Compared to adolescents without diabetes, those with diabetes had a higher prevalence of hypertensive disorders (T1D: 35.4%, T2D: 37.8%, GDM: 24.9%, None: 9.9%; *p* <0:001) and preterm labor (T1D: 21.5%, T2D: 16.8%, GDM: 6.8%, none: 5.7%; *p* <0:001). In adjusted models, odds of hypertensive disorders were higher in later study years (2019 vs. 2006 OR 1.85, 95% CI 1.77–1.94), among those with T1D (OR 4.32, 95% CI 3.94–4.74), with T2D (OR 4.18, 95% CI 3.79–4.61), and with GDM (OR 1.99, 95% CI 1.89–2.10). Adjusted odds of preterm labor were higher among those with T1D (OR 4.53, 95% CI 4.09–5.02), with T2D (OR 3.35, 95% CI 2.96–3.78), and with GDM (OR 1.18, 95% CI 1.08–1.28); disparities were seen by race/ethnicity, insurance, and income.

**Conclusions:**

Diabetes, which is increasing among adolescents, is a significant risk factor for preterm labor and hypertensive disorders. Though the absolute number of adolescent pregnancies is decreasing, rates of hypertensive disorders have increased. Appropriate interventions are needed to ensure healthy outcomes for adolescents who are pregnant.

## 1. Introduction

Adolescent pregnancies, defined in the literature as occurring between ages 10 and 19, occurred in 16.7 per 1000 females and 4.6% of all live births in the United States in 2019 [1]. Although adolescent pregnancy childbearing rates have seen a dramatic decline since the 1990s, these rates remain substantially higher in the United States than other industrialized nations [1, 2]. Contributors to adolescent birth rates include racial and/or ethnic disparities, geopolitical influences on access to effective contraception and family planning, and other social determinants of health [3, 4].

Adolescent pregnancies are associated with increased rates of maternal and fetal complications, such as hypertensive disorders of pregnancy (including eclampsia), preterm birth, blood transfusions, congenital birth defects, low APGAR scores, suspected neonatal sepsis, and neonatal assisted ventilation [5]. The intersection of diabetes and adolescent pregnancy warrants even further concern. The diabetes epidemic has exponentially increased over the past few decades, with pregestational (type 1 and type 2) diabetes affecting 3.1%–6.8% of reproductive-aged women and 1%–2% of pregnancies in the United States. In 2019, an estimated 283,000 children and adolescents younger than age 20 years—or 35 per 10,000 US youths—had diagnosed diabetes [6]. Like adolescent pregnancies, diabetes in pregnancy is also associated with adverse maternal and fetal outcomes, including miscarriage, congenital fetal anomalies, preterm delivery, hypertensive disorders of pregnancy, fetal macrosomia, cesarean delivery, neonatal complications, maternal hyperglycemia, and worsening diabetic retinopathy and nephropathy [7].

Although the rates of gestational diabetes (GDM) in adolescent pregnancies have been explored, there is a paucity of data on the combination of pregestational diabetes and adolescent pregnancies, which is a vulnerable and high-risk subset of the obstetrics population in the United States. The objective of this study is threefold: (1) to describe the obstetrical risks of two common diabetes complications (hypertensive disorders and preterm labor with delivery) and determine how these outcomes are modified by diabetes status and type, (2) to evaluate what other patient and clinical factors predict increased risk for these serious complications in this understudied population, and (3) to evaluate the health expenditures associated with these complicated delivery admissions.

## 2. Methods

### 2.1. Study Population and Data Elements.

We used the Healthcare Cost and Utilization Project (HCUP) Kids’ Inpatient Database (KID) developed by the Agency for Healthcare Research and Quality (AHRQ) [8]. As previously described [9], KID is a publicly available deidentified database containing admissions from children and youth ≤20 years old (yo) from 42,000 hospitals across 46 states, sampled 80% of nonnewborn admissions. KID data are available every 3 years, but 2015 data were not released, and instead, 2016 data were released due to the transition from International Code of Diseases (ICD)-9 to ICD-10 coding.

We identified labor and delivery admissions in years 2006, 2009, 2012, 2016, and 2019 using ICD codes for normal delivery and other indications for care in labor and delivery (ICD-9 codes 650–659 and ICD-10 codes O61-O77, O80, and O82). We classified patients as having type 1 diabetes (T1D; ICD-9: 250.X1, 250.X3, ICD-10: E10), type 2 diabetes (T2D; ICD-9: 250.X0, 250.X2, ICD-10: E11), GDM (ICD-9: 648.0 without codes for T1D or T2D, ICD-10: O244), or having no diabetes (none of the aforementioned codes). Our clinical outcomes of hypertensive disorders included diagnoses of hypertension (HTN) complicating pregnancy childbirth and the puerperium (ICD-9: 642), pre-existing HTN complication pregnancy childbirth and the puerperium (ICD-10: O10), pre-existing HTN with pre-eclampsia (ICD-10: O11), pregestational HTN without significant proteinuria (ICD-10: O13), pre-eclampsia (ICD-10: O14), eclampsia (ICD-10: O15), and unspecified maternal HTN (ICD-10: O16). Preterm labor with delivery, henceforth referred to as preterm labor, was defined as a diagnosis for early onset of delivery, delivered with or without mention of antepartum condition (ICD-9: 644.21), and preterm labor with preterm delivery (ICD-10: O601). We adjusted our analyses for several patient level (e.g., age, race/ethnicity, income, comorbidities, and urban residence) and hospital level (e.g., region, size, and ownership) covariates. The definitions of these factors were described in a former publication [9]. Severity of illness on admission was determined using All Patient Refined Diagnosis-Related Groups, [10] which classifies patients according to their reasons for admission, severity of illness, risk of mortality, resource intensity, and disposition

### 2.2. Statistical Analysis.

Weights provided by KID were used to generate nationally representative estimates of hospital admissions and all analyses accounted for the stratified sampling design. We used descriptive statistics to summarize the characteristics of all labor and delivery admissions. Counts less than or equal to 10 are noted as ≤10 per HCUP policy to preserve the amenity and privacy rights of those individuals. A chi-squared test was used to evaluate for unadjusted statistical differences in demographics, clinical features, and between diabetes status and type. We performed logistic regression to evaluate the adjusted association of diabetes status and type and hypertensive disorders and preterm labor after adjusting for confounding factors. All analyses were performed with Stata version 15.1 (StataCorp, College Station, TX). We retained missing/unknown categories for all variables with missing data, resulting in a consistent sample across all analyses. This study was institutional review board (IRB) exempt as it was a secondary analysis of pre-existing and deidentified data. This report follows the strengthening the reporting of observational studies in epidemiology (STROBE) reporting guidelines for cross-sectional studies.

## 3. Results

### 3.1. Characteristics of Labor and Delivery Admissions by Diabetes Type.

There were 1,843,139 labor and delivery admissions across our study years, and the number of admissions decreased over time from 477,044 in 2006 to 251,439 in 2019 (Table 1). These admissions occurred most commonly in those >15 yo (98%) of non-Hispanic White race (35.6%), with public insurance (72.4%), living in urban areas (80.4%), of the lowest income quartile (41.2%), and in hospitals in the southern United States (44.5%). Of these admissions, 3327 (0.2%) had T1D, 3050 (0.2%) had T2D, and 12,909 (0.7%) had GDM. Pregnancies in the youngest age group (9–11 yo) occurred only in patients without diabetes. The racial–ethnic breakdown of admissions varied by diabetes type. Admissions with T1D and GDM occurred more commonly in those who were non-Hispanic White. T2D occurred similarly and most frequently in those who were non-Hispanic Black and non-Hispanic White race. Across all diabetes types, admissions occurred more prevalently in those with public insurance, with income in the lower two income quartiles, living in urban areas, and living in southern regions.

Labor and delivery admissions were more complicated, lengthy, and costly in patients with diabetes compared to those without diabetes. Patients without diabetes more often had admissions classified as having a minor severity of illness (60%), while those with GDM had primarily a moderate severity of illness (62.3%), and those with T1D and T2D had a major severity of illness (56.3% and 58.8%, respectively). The mean length of stay and admission charge were 2.59 days (95% CI 2.58–2.60) and $15,316 ($15,113–$15,517) in those without diabetes and highest in those with T1D at 4.81 days (4.58–5.04 days) and $29,279 ($27,942–$30,616). Those with diabetes were most likely to have comorbid conditions such as obesity, renal disease, multiple gestations, and hospital complications such as fetal abnormality and premature rupture of membranes (PROM; Table 2). Delivery by cesarean section occurred at a frequency of 19% in the general population. Among those with diabetes, cesareans occurred most frequently in those with T1D (50.6%), followed by T2D (42.7%) and GDM (27.3%).

### 3.2. Hypertensive Disorders.

Hypertensive disorders were observed in 10% of labor and delivery admissions and were common among adolescents with T2D (37.8%), followed by those with T1D (35.4%) and GDM (24.9%). Those with hypertensive disorders were more likely to be obese (10.9% vs. 3.1%), have renal disease (0.4% vs. 0%), multiple gestations (1.2% vs. 0.5%), and fetal abnormality (1.5% vs. 1.2%). PROM occurred less in those with hypertensive disorders (3.5% vs. 4.7%) (Table 3). C-sections occurred in 33.5% with hypertensive disorder compared to 14.4% in those without. Length of stay and hospital charge were higher in those with a hypertensive disorder (3.60 [95% CI 3.58–3.63] and $22,784 [$22,422–23,145] vs. 2.49 days [95% CI 248–2.50] and $14,596 [95% CI $14,404–$14,7882]).

In adjusted models, the odds of hypertensive disorders increased over time, with a 1.85 higher odds in 2019 as compared to 2006 (95% CI 1.77–1.94, *p* <0:001; Table 4). As compared to adolescents without diabetes, those with T1D had the highest odds of hypertensive disorder (OR 4.32, 95% CI 3.91–4.74, *p* <0:001), followed by T2D (OR 4.18, 95% CI 3.79–4.61, *p* <0:001) and GDM (OR 1.99, 95% CI 1.89–2.10, *p* <0:001). Other notable predictors of hypertensive disorders were being in the age group 12–14 yo compared to 18–20 yo (OR 1.29, 95% CI 1.22–1.36, *p* <0:001) and being of Black race (OR 1.26, 95% CI 1.23–1.30, *p* <0:001) or Native American race (OR 1.15, 95% CI 1.05–1.23, *p* <0:003) compared to the White race. Those of Hispanic and Asian ancestry had lower odds of hypertensive disorder (OR 0.83, 0.81–0.85, *p* <0:001, and OR 0.76, 0.68–0.84, *p* <0:001, respectively). Patients of the lowest income quartile had higher odds for hypertensive disorder compared to the highest income quartile (OR 1.06, 95% CI 1.03–1.10, *p* <0:001). Those with multiple gestations (OR 2.33, 95% CI 2.19–2.47, *p* <0:001), obesity (OR 3.19, 95% CI 3.11–3.28, *p* <0:001), and renal disease (OR 6.59, 95% CI 5.76–7.55, *p* <0:001) were also at increased odds for a hypertensive disorder. We performed a sensitivity analysis to evaluate for differences in the predictors of severe hypertensive disorders (e.g., eclampsia, HELLP [hemolysis, elevated liver enzymes and low platelets], and pre-existing HTN with superimposed pre-eclampsia) compared to milder hypertensive disorder (e.g., mild transient HTN, mild pre-eclampsia, etc.) and found a similar trend as previously noted with patients with T1D possessing the highest risk (OR 2.19, 95% CI 1.88–2.54).

### 3.3. Preterm Labor.

Preterm labor occurred in 5.8% of pregnancies, and those with T1D had the highest proportion of preterm labor at 21.5%, followed by T2D (16.8%) and GDM (6.8%). Those with preterm labor were more likely to have multiple gestations (4.3% vs. 0.3%), PROM (14.5% vs. 4%), and fetal abnormality (2.9% vs. 1.1%). C-sections occurred in 25.7% of deliveries with preterm labor compared to 18.6% in those without. Length of stay and hospital charge were higher in those with preterm labor (3.72 days [95% CI 3.67–3.77] and $20,784 [95% CI $20,410–21,157] vs. 2.54 days [95% CI 2.53–2.55] and $15,091 [95% CI $14,892–$15,290]).

In adjusted models, the odds of preterm labor varied over time but trended upward with the odds of preterm in 2016 (OR 1.15, 95% CI 1.10–1.2, *p* <0:001) and 2019 (OR 1.07, 95% CI 1.03–1.12, <*p=*0:001) being higher than in 2006. Patients with T1D had the highest odds for preterm labor (OR 4.53, 95% CI 4.09–5.02), followed by T2D (OR 2.96, 95% CI 2.96–3.78, *p* <0:001) and GDM (OR 1.18, 95% CI 1.08–1.28, *p* <0:001) compared to those without diabetes. Other notable predictors of preterm labor include age 12–14 (OR 1.77, 95% CI 1.66–1.88, *p* <0:001) compared to 18–20 yo and being of Black race (OR 1.27 (OR 1.24−1.31, *p* <0:001) or Asian race (OR 1.23, OR 1.15–1.32, *p* <0:001). Those of Hispanic ethnicity were at lower risk of preterm labor (OR 0.94, 95% CI 0.92–0.97, *p* <0:001). Patients without private insurance (e.g., public, self-pay, and others) and those in the lowest two income quartile (compared to highest income quartile) also had higher odds of preterm labor. Lastly, those with multiple gestations (OR 14.91, 95% CI 14.21–15.71, *p* <0:001) and renal disease (OR 2.36, 95% CI 1.96–2.83, *p* <0:001) had increased odds for preterm labor, while those with obesity had lower odds (OR 0.79 95% CI 0.75–0.83, *p* <0:001).

## 4. Discussions

While we found that adolescent pregnancies have decreased over the past two decades, our study shows concerning rises in odds of both hypertensive disorders in pregnancy and preterm labor. The decreasing frequency of adolescent pregnancies over the time period analyzed matches the temporal trends described previously in the literature [1]. However, social and geographic determinants of health, including public insurance, lower socioeconomic status, urban locale, and location in Southern states of the United States, continue to be associated with adolescent pregnancies in those with and without diabetes. These data suggest progress toward the Healthy People 2030 goal of reducing pregnancies in adolescents but not of the overarching goal of eliminating disparities and achieving health equity [11]. A root cause analysis must be conducted to determine the barriers to decreasing adolescent birth rates in these populations [12].

Adolescent pregnancies are at inherently higher risk of adverse pregnancy outcomes, and this risk is further potentiated by concomitant diabetes. We report higher odds of multiple maternal and fetal sequelae, including fetal anomaly, preterm delivery, hypertensive disorders, cesarean delivery, and increased costs of care during the labor and delivery admission, when compared to adolescent pregnancies without diabetes. The estimated costs of adolescent pregnancies are likely further compounded when the adolescents enter pregnancy with a comorbid diabetes diagnosis.

The prevalence of diabetes in youth has increased significantly in the last two decades and continues to rise in recent years [13, 14]. While the pregnancies in persons with diabetes were relatively stable during this study, in the setting of decreasing rates of overall adolescent deliveries, this is concerning for a potential increase in pregnancies in those with diabetes over time, which may result in increased pregnancy complications (e.g., hypertensive disorders and preterm labor) in this population. Additionally, rising rates of obesity are likely to increase the risk of some pregnancy complications. This may not only have short-term implications but also impact long-term outcomes given the association of future cardiovascular disease in women who have hypertensive disorders during pregnancy [15]. We found that obesity was associated with lower rate of preterm labor. This is consistent with existing literature showing that obese mothers are more likely to have post-term birth than preterm birth [16].

Preconception optimization of diabetes care and reproductive planning discussions continue to be suboptimal in all populations but particularly so in adolescents [17, 18]. These findings may be translated as a call to arms for pediatricians and endocrinologists managing adolescents with diabetes to be trained in contraception and reproductive health counseling and to include gynecologists and maternal–fetal medicine specialists in the multidisciplinary management of this population.

This study has some limitations, including those inherent to retrospective studies involving databases derived from administrative coding. This includes misclassification of diabetes type or pregnancy complications and the inability to account for change in coding practices in the setting of ICD9 to ICD-10 change, which, for example, may account for the significant increase in case detection in GDM after transition to ICD-10. Furthermore, we were unable to pair data on the neonates resulting from these pregnancies, which carry additional public health and societal implications. Despite these limitations, this study captures the most recent estimate of adolescent pregnancies suing the largest national population-based database of pediatric discharges, which strengthen the ability to study this specific high-risk subpopulation of adolescent pregnancies. In addition to describing the associated clinical outcomes, we also evaluated the healthcare costs associated with the comorbid pregnancies during the labor and delivery admission. Future work will focus on the impact duration of diabetes, glycemic control, approach to diabetes management, and how they modify pregnancy outcomes in this population.

## 5. Conclusions

Pregnancy in adolescence is associated with higher rates of complications, and when compounded by a diagnosis of diabetes, these risks increase further. Given the rising rates of diabetes in this age group, better strategies are needed to improve reproductive planning discussions and prenatal optimization in this population and high risk for poor outcomes.

## Figures and Tables

**TABLE 1:** Patient and hospital characteristics of labor and delivery admissions by diabetes type.

Characteristic	No diabetes	(%)	Type 1	(%)	Type 2	(%)	Gestational DM	(%)	Total	%	*p*-Value

*n*	1,823,854	—	3327	—	3050	—	12,909	—	1,843,139	—	—
Age											<0.001
9–11	381	0.0%	0	0.0%	0	0.0%	0	0.0%	381	0.0%	—
12–14	18,866	1.0%	25	0.8%	20	0.7%	35	0.3%	18,946	1.0%	—
15–17	377,105	20.7%	554	16.6%	392	12.9%	1397	10.8%	379,448	20.6%	—
18–20	1,425,279	78.1%	2748	82.6%	2635	86.4%	11,476	88.9%	1,442,138	78.2%	—
Unknown	2223	0.1%	0	0.0%	<10	0.1%	0	0.0%	2226	0.1%	—
Race/ethnicity											
White	648,103	35.5%	1593	47.9%	857	28.1%	5557	43.0%	656,110	35.6%	<0.001
Black	338,730	18.6%	628	18.9%	878	28.8%	1966	15.2%	342,202	18.6%	—
Hispanic	495,136	27.1%	599	18.0%	754	24.7%	3790	29.4%	500,280	27.1%	—
Asian or Pacific Islander	24,413	1.3%	29	0.9%	34	1.1%	249	1.9%	24,724	1.3%	—
Native American	17,546	1.0%	27	0.8%	48	1.6%	269	2.1%	17,890	1.0%	—
Other	72,664	4.0%	95	2.8%	108	3.5%	501	3.9%	73,367	4.0%	—
Unknown	227,262	12.5%	357	10.7%	371	12.2%	576	4.5%	228,566	12.4%	—
Payer											
Private	397,232	21.8%	963	29.0%	561	18.4%	2927	22.7%	401,684	21.8%	—
Public	1,321,010	72.4%	2197	66.0%	2310	75.8%	9486	73.5%	1,335,002	72.4%	—
Self-pay	55,428	3.0%	40	1.2%	85	2.8%	202	1.6%	55,755	3.0%	—
Other/unknown	50,185	2.8%	126	3.8%	93	3.1%	294	2.3%	50,698	2.8%	—
Urbanicity											<0.001
Urban areas	1,466,140	80.4%	2586	77.7%	2450	80.3%	10,265	79.5%	1,481,440	80.4%	—
Nonurban areas	348,293	19.1%	725	21.8%	589	19.3%	2629	20.4%	352,236	19.1%	—
Household income											
Quartile 4	164,768	9.0%	316	9.5%	236	7.8%	1063	8.2%	166,384	9.0%	<0.001
Quartile 3	364,165	20.0%	700	21.1%	479	15.7%	2625	20.3%	367,969	20.0%	—
Quartile 2	509,169	27.9%	1002	30.1%	796	26.1%	3782	29.3%	514,749	27.9%	—
Quartile 1	751,451	41.2%	1245	37.4%	1492	48.9%	5300	41.1%	759,489	41.2%	—
Unknown	34,301	1.9%	63	1.9%	46	1.5%	138	1.1%	34,548	1.9%	—
Region of hospital											
Northeast	211,404	11.6%	429	12.9%	298	9.8%	1450	11.2%	213,581	11.6%	<0.001
Midwest	386,347	21.2%	805	24.2%	618	20.3%	2901	22.5%	390,671	21.2%	—
South	811,250	44.5%	1446	43.5%	1551	50.9%	5648	43.8%	819,895	44.5%	—
West	414,853	22.7%	647	19.5%	582	19.1%	2910	22.5%	418,993	22.7%	—
Hospital ownership											<0.001
Private	1,322,124	72.5%	2351	70.7%	2087	68.4%	10,753	83.3%	1,337,314	72.6%	—
Public	501,730	27.5%	976	29.3%	963	31.6%	2156	16.7%	505,825	27.4%	—
Hospital size											<0.001
Large	1,073,768	58.9%	2236	67.2%	2015	66.1%	7268	56.3%	1,085,287	58.9%	—
Medium	486,904	26.7%	743	22.3%	700	22.9%	3337	25.9%	491,684	26.7%	—
Small	239,815	13.1%	287	8.6%	285	9.4%	2295	17.8%	242,682	13.2%	—
Unknown	23,367	1.3%	60	1.8%	49	1.6%	<10	0.1%	23,485	1.3%	—
Year											<0.001
2006	475,604	26.1%	621	18.7%	659	21.6%	160	1.2%	477,044	25.9%	—
2009	454,795	24.9%	634	19.0%	722	23.7%	124	1.0%	456,275	24.8%	—
2012	359,306	19.7%	675	20.3%	655	21.5%	99	0.8%	360,736	19.6%	—
2016	290,039	15.9%	752	22.6%	534	17.5%	6321	49.0%	297,645	16.1%	—
2019	244,110	13.4%	645	19.4%	480	15.7%	6204	48.1%	251,439	13.6%	—

**TABLE 2:** Clinical characteristics of labor and delivery admissions by diabetes type.

Characteristic	No diabetes	(%)	Type 1	(%)	Type 2	(%)	Gestational	(%)	Total	(%)	*p*-Value

*n*	1,823,854	99.0%	3327	0.2%	3050	0.2%	12,909	0.7%	1,843,139	100%	—
Preterm labor	104,246	5.7%	717	21.5%	513	16.8%	875	6.8%	106,351	5.8%	<0.001
Hypertensive disorders	179,982	9.9%	1177	35.4%	1152	37.8%	3214	24.9%	185,525	10.1%	<0.001
Multiple gestations	9553	0.5%	15	0.5%	22	0.7%	135	1.1%	9726	0.5%	<0.001
PROM	83,728	4.6%	240	7.2%	172	5.6%	940	7.3%	85,080	4.6%	<0.001
Premature PROM^b^	16,891	0.9%	116	3.5%	61	2.0%	427	3.3%	17,494	0.9%	<0.001
Fetal abnormality	22,085	1.2%	144	4.3%	95	3.1%	154	1.2%	22,478	1.2%	<0.001
Obesity	68,462	3.8%	303	9.1%	701	23.0%	2582	20.0%	72,049	3.9%	<0.001
Renal disease	1327	0.1%	68	2.1%	34	1.1%	33	0.3%	1462	0.1%	<0.001
C-section	343,866	18.9%	1685	50.6%	1302	42.7%	3526	27.3%	350,379	19.0%	<0.001
Died	75	0.0%	<10	0.0%	<10	0.1%	<10	0.0%	79	0.0%	0.4286
Severity of illness											<0.001
No class	274	0.0%	<10	0.0%	<10	0.0%	<10	0.0%	278	0.0%	—
Minor	1,101,877	60.4%	41	1.2%	36	1.2%	2894	22.4%	1,104,848	59.9%	—
Moderate	614,776	33.7%	1303	39.2%	1155	37.9%	8044	62.3%	625,279	33.9%	—
Major	102,977	5.7%	1873	56.3%	1794	58.8%	1911	14.8%	108,554	5.9%	—
Extreme	3950	0.2%	109	3.3%	64	2.1%	58	0.4%	4180	0.2%	—
LOS (days)^a^	2.59	2.58–2.60	4.81	4.58–5.04	4.23	3.99–4.46	3.30	3.23–3.80	1.60	2.59–2.62	—
Total charges (2019 dollars)^a^	$15,316	$15,113– $15,517	$29,279	$27,942–$30,616	$25,237	$23,946–$26,527	$24,160	$23,430–$24,890	$15,420	15,215–15,623	—

a Mean reported with 95% confidence interval.

b Prevalence reported for only years 2016 and 2019.

**TABLE 3:** Clinical characteristics of pregnancies complicated by hypertensive disorder or preterm labor.

Characteristic		Hypertensive disorder				Preterm labor		
	NO	(%)	YES	(%)	NO	(%)	YES	(%)

*N*	1,657,614	—	185,525	—	1,736,788	—	106,351	—
Obesity	51,755	3.1%	20,293	10.9%	68,434	3.9%	3615	3.4%
Renal disease	733	0.0%	729	0.4%	1241	0.1%	221	0.2%,
Multiple gestations	7503	0.5%	2222	1.2%	5118	0.3%	4608	4.3%
PROM	78,636	4.7%	6445	3.5%	69,681	4.0%	15,399	14.5%
Premature PROM^b^	183,787	99.1%	1738	0.9%	17,494	0.9%	97,199	91.4%
Fetal abnormality	19,702	1.2%	2776	1.5%	19,399	1.1%	3079	2.9%
Cesarean section	288,204	17.4%	62,174	33.5%	322,951	18.6	27,427	25.8%
LOS^a^	2.49	2.48–2.50	3.60	3.58–3.63	2.54	2.53–2.55	3.72	3.67–3.77
Total charges^a^(2019 dollars)	$14,596	$14,404-$ 14,788	$22,784	$ 22,422—$23,145	$15,091	$14,892—$15,290	$20,784	$20,410—$21,157

Abbreviations: LOS, length of stay; PROM, premature rupture of membranes.

a Mean and 95% confidence interval reported.

b Prevalence reported for only years 2016 and 2019.

**TABLE 4:** Odds of preterm labor and hypertensive disorder.

Variable		Preterm labor				Hypertensive disorder		
	OR	95% CI		*p*-Value	OR	95% CI		*p*-Value

Age								
9–11	0.21	0.07	0.66	0.008	0.34	0.16	0.72	0.005
12–14	1.77	1.66	1.88	<0.001	1.29	1.22	1.36	<0.001
15–17	1.15	1.12	1.17	<0.001	1.07	1.05	1.08	<0.001
18–20		Reference				Reference		
Unknown	1.17	0.92	1.49	0.194	0.69	0.52	0.92	0.010
Race/ethnicity								
White		Reference				Reference		
Black	1.27	1.24	1.31	<0.001	1.26	1.23	1.30	<0.001
Hispanic	0.94	0.92	0.97	<0.001	0.83	0.81	0.85	<0.001
Asian or Pacific Islander	1.23	1.15	1.32	<0.001	0.76	0.68	0.84	<0.001
Native American	1.08	0.98	1.18	0.120	1.15	1.05	1.26	0.003
Other/unknown	1.05	1.00	1.09	0.039	0.95	0.91	0.99	0.006
Diabetes status and type								
No diabetes		Reference				Reference		
Type 1	4.53	4.09	5.02	<0.001	4.32	3.94	4.74	<0.001
Type 2	3.35	2.96	3.78	<0.001	4.18	3.79	4.61	<0.001
Gestational diabetes	1.18	1.08	1.28	<0.001	1.99	1.89	2.10	<0.001
Co-existing conditions^a^								
Multiple gestations	14.94	14.21	15.71	<0.001	2.33	2.19	2.47	<0.001
Obesity	0.79	0.75	0.83	<0.001	3.19	3.11	3.28	<0.001
Renal disease	2.36	1.96	2.83	<0.001	6.59	5.76	7.55	<0.001
Payer								
Private		Reference				Reference		
Public	1.03	1.01	1.06	0.002	0.94	0.92	0.96	<0.001
Self-pay or no charge	1.23	1.17	1.30	<0.001	0.82	0.77	0.87	<0.001
Other/unknown	1.12	1.06	1.19	<0.001	0.95	0.91	1.00	0.036
Household income								
Quartile 1	1.09	1.05	1.13	<0.001	1.06	1.03	1.10	<0.001
Quartile 2	1.04	1.01	1.08	0.023	1.01	0.98	1.04	0.657
Quartile 3	1.01	0.98	1.05	0.371	1.01	0.98	1.04	0.350
Quartile 4		Reference				Reference		
Unknown	1.05	0.97	1.12	0.217	1.09	1.00	1.19	0.048
Urbanicity								
Nonurban areas	0.96	0.93	0.99	0.004	0.91	0.89	0.94	<0.001
Urban areas		Reference				Reference		
Unknown	1.09	0.93	1.28	0.274	0.98	0.86	1.11	0.698
Region of hospital								
Northeast		Reference				Reference		
Midwest	1.03	0.98	1.08	0.306	1.00	0.95	1.06	0.904
South	1.04	1.00	1.09	0.055	1.07	1.02	1.12	0.009
West	0.97	0.92	1.01	0.161	0.91	0.86	0.96	<0.001
Hospital ownership								
Public	1.13	1.09	1.17	<0.001	1.22	1.17	1.27	<0.001
Private		Reference				Reference		
Hospital size								
Small	0.76	0.73	0.80	<0.001	0.87	0.84	0.91	<0.001
Medium	0.89	0.86	0.92	<0.001	0.91	0.87	0.94	<0.001
Large		Reference				Reference		
Unknown	1.29	1.12	1.48	<0.001	1.24	1.09	1.41	0.001
Year								
2006		Reference				Reference		
2009	1.00	0.97	1.04	0.930	1.13	1.09	1.17	<0.001
2012	0.95	0.91	0.99	0.017	1.19	1.13	1.25	<0.001
2016	1.15	1.10	1.20	<0.001	1.38	1.32	1.44	<0.001
2019	1.07	1.03	1.12	0.001	1.85	1.77	1.94	<0.001

*Note:* Variables included in this adjusted model include age, race/ethnicity, diabetes status/type, select co-existing conditions (multiple gestations, obesity, and renal disease), payer, household income, urbanity, region of the hospital, hospital ownership, hospital size, and year.

a Variables listed under co-existing conditions are all binary variables (condition present vs. absent), so the reference level is condition absent, but this has been omitted from the table for brevity.

## Data Availability

The data that support the findings of this study are available from the Agency for healthcare Research and Quality’s (AHRQ) Healthcare Cost and Utilization Project (HCUP). Restrictions apply to the availability of these data, which were used under license for this study.
